# Supplementary material for: Population Genomic Evidence for the Diversification of Bellamya aeruginosa in Different River Systems in China
Source: Biology (Basel). 2022 Dec 23;12(1):29. doi: 10.3390/biology12010029 (PMC9855799; doi:10.3390/biology12010029)
Supplement: Supplementary file 1 [file biology-12-00029-s001.zip › Table S1-3.pdf]

Table S1 The high-quality base ratio (Q30) and GC percentage for each *B. aeruginosa* accessions and the control, as well as the GenBank accession number for gene mtDNA *COI* generated in present study.

Accession	*COI*	BMK ID	Total Reads	Q30 Percentage (%)	GC Percentage (%)
YW3	OP889150	aa	221,633	91.55	36.64
YW9	OP889151	ab	352,141	87.91	37.71
YW10	OP889152	ac	284,250	91.60	36.62
YW42	OP889153	ad	179,449	91.88	36.71
YW43	OP889154	ae	283,387	91.76	36.90
YW14	OP889155	bu	148,547	90.23	41.30
YW15	OP889156	bv	244,529	90.42	39.23
YW16	OP889157	bw	205,770	90.15	40.33
YW37	OP889158	af	218,236	91.86	36.98
YW39	OP889159	ag	224,360	91.54	36.94
YW46	OP889160	bx	250,170	90.18	40.16
YC4	OP889161	bb	234,718	89.77	39.85
YC5	OP889162	bc	258,209	89.97	39.97
YC7	OP889163	bd	327,820	90.07	39.78
YC8	OP889164	be	276,408	89.97	39.31
YC9	OP889165	bf	301,109	90.26	39.65
YC10	OP889166	bg	274,745	90.04	39.40
YC11	OP889167	bh	335,599	90.17	39.36
YC12	OP889168	bi	267,311	89.94	40.08
YC15	OP889169	bj	253,549	89.53	40.24
YC16	OP889170	bk	189,159	89.67	40.35
YC20	OP889171	bl	185,590	90.20	39.77
YC24	OP889172	bm	180,087	90.28	40.16
LZH2	OP889173	bn	224,242	90.07	39.04
LZH5	OP889174	bo	255,095	90.31	38.72
LZH6	OP889175	bp	253,574	90.01	38.82
LZH10	OP889176	bq	243,078	89.42	38.97
LZH13	OP889177	br	274,022	89.81	38.77
LZH15	OP889178	bs	279,515	90.58	38.47
LZH16	OP889179	bt	214,085	90.07	39.16
YH1	OP889180	by	257,212	90.28	38.55
YH2	OP889181	bz	260,618	90.24	38.89
YH4	OP889182	ca	321,772	90.37	38.48
YH9	OP889183	cb	289,857	90.06	38.76
YH10	OP889184	cc	282,311	90.02	38.52
YH13	OP889185	cd	274,988	90.11	38.80
YH14	OP889186	ce	279,090	90.20	38.00
YH15	OP889187	cf	260,335	90.18	38.18
YH19	OP889188	dj	259,695	90.33	39.70
YH24	OP889189	dk	244,888	90.13	38.92

YH26	OP889190	dl	137,417	89.40	40.20
YH28	OP889191	dm	222,628	89.99	39.27
WL4	OP889192	ch	287,556	89.65	41.70
WL5	OP889193	ci	213,482	89.50	40.84
WL6	OP889194	cj	249,855	89.40	40.22
WL7	OP889195	ck	203,470	89.79	40.90
WL18	OP889196	cl	194,597	89.47	38.09
WL20	OP889197	cm	304,667	89.72	40.02
WL23	OP889198	cn	288,084	89.80	39.00
WL24	OP889199	co	305,023	89.66	41.24
QSH3	OP889200	cp	286,473	89.69	39.65
QSH4	OP889201	cq	283,162	89.66	39.59
QSH 8	OP889202	cr	266,355	90.25	38.78
QSH 9	OP889203	cs	302,267	90.06	39.27
QSH 10	OP889204	ct	253,004	89.95	41.01
QSH18	OP889205	cv	288,883	90.01	40.12
QSH20	OP889206	cx	262,906	89.53	40.29
LB1	OP889207	cy	294,800	90.61	39.36
LB2	OP889208	cz	279,075	89.80	38.98
LB3	OP889209	da	328,294	90.14	39.17
LB5	OP889210	db	324,792	90.08	39.44
LB6	OP889211	dc	209,483	90.12	41.29
LB7	OP889212	dd	283,085	90.46	39.89
LB8	OP889213	de	272,817	88.05	39.12
LB9	OP889214	df	278,624	89.79	39.43
LB10	OP889215	dg	258,610	90.00	41.09
LB11	OP889216	dh	231,101	89.80	40.13
LB12	OP889217	di	290,298	89.95	39.85
Rice		Control	109,008	87.54	46.67

Table S2 The number and SLAF-seq depth of each *B. aeruginosa* accessions

**Sample ID**	**BMK ID**	**SLAF number**	**Total depth**	**Average depth**
YW3	aa	12,248	158,915	12.97
YW9	ab	12,663	179,516	14.18
YW10	ac	12,816	201,426	15.72
YW42	ad	11,880	127,500	10.73
YW43	ae	12,911	191,751	14.85
YW14	bu	11,506	86,046	7.48
YW15	bv	12,922	165,143	12.78
YW16	bw	12,289	124,496	10.13
YW37	af	11,948	151,472	12.68
YW39	ag	12,045	152,619	12.67
YW46	bx	12,615	175,998	13.95
YC4	bb	12,683	169,514	13.37

YC5	bc	12,904	188,714	14.62
YC7	bd	13,045	226,222	17.34
YC8	be	12,882	197,473	15.33
YC9	bf	12,940	207,888	16.07
YC10	bg	12,885	198,828	15.43
YC11	bh	13,323	242,396	18.19
YC12	bi	12,692	192,642	15.18
YC15	bj	12,731	172,558	13.55
YC16	bk	11,737	114,863	9.79
YC20	bl	11,670	129,583	11.10
YC24	bm	11,475	121,816	10.62
LZH2	bn	12,531	158,050	12.61
LZH5	bo	13,196	176,073	13.34
LZH6	bp	12,657	178,744	14.12
LZH10	bq	12,872	167,740	13.03
LZH13	br	13,075	193,436	14.79
LZH15	bs	13,279	198,285	14.93
LZH16	bt	12,858	147,819	11.50
YH1	by	12,971	182,767	14.09
YH2	bz	12,950	191,473	14.79
YH4	ca	13,443	210,700	15.67
YH9	cb	13,411	209,124	15.59
YH10	cc	13,298	193,517	14.55
YH13	cd	13,133	197,303	15.02
YH14	ce	13,395	197,740	14.76
YH15	cf	12,863	189,889	14.76
YH19	dj	12,955	188,733	14.57
YH24	dk	13,242	165,171	12.47
YH26	dl	12,402	87,994	7.10
YH28	dm	13,005	152,786	11.75
WL4	ch	12,818	159,835	12.47
WL5	ci	12,537	135,738	10.83
WL6	cj	13,150	168,172	12.79
WL7	ck	12,647	130,080	10.29
WL18	cl	12,129	141,825	11.69
WL20	cm	13,357	216,431	16.20
WL23	cn	13,018	208,873	16.04
WL24	co	13,209	196,948	14.91
QSH3	cp	13,046	192,823	14.78
QSH4	cq	13,174	197,726	15.01
QSH 8	cr	13,069	181,713	13.90
QSH 9	cs	13,347	214,625	16.08
QSH 10	ct	12,629	164,822	13.05
QSH18	cv	13,055	201,140	15.41

QSH20	cx	12,972	173,044	13.34
LB1	cy	13,421	203,048	15.13
LB2	cz	13,274	199,864	15.06
LB3	da	13,639	233,259	17.10
LB5	db	13,668	221,765	16.23
LB6	dc	12,141	135,388	11.15
LB7	dd	13,156	186,311	14.16
LB8	de	13,180	197,287	14.97
LB9	df	13,425	195,840	14.59
LB10	dg	13,027	172,589	13.25
LB11	dh	12,509	161,767	12.93
LB12	di	13,040	165,738	12.71

Table S3 Detailed information and the admixture coefficients at $K = 3$ for each accession of *B. aeruginosa*

Accession	BMK ID	Locality	River system	Gree cluster	Yellow cluster	Gray cluster
YW3	aa	Xianning, Hubei, CN	Yangtze R.	0.99998	0.00001	0.00001
YW9	ab	Xianning, Hubei, CN	Yangtze R.	0.99998	0.00001	0.00001
YW10	ac	Xianning, Hubei, CN	Yangtze R.	0.99998	0.00001	0.00001
YW42	ad	Xianning, Hubei, CN	Yangtze R.	0.99998	0.00001	0.00001
YW43	ae	Xianning, Hubei, CN	Yangtze R.	0.99998	0.00001	0.00001
YW14	bu	Xianning, Hubei, CN	Yangtze R.	0.99998	0.00001	0.00001
YW15	bv	Xianning, Hubei, CN	Yangtze R.	0.99998	0.00001	0.00001
YW16	bw	Xianning, Hubei, CN	Yangtze R.	0.99998	0.00001	0.00001
YW37	af	Xianning, Hubei, CN	Yangtze R.	0.00001	0.00001	0.99998
YW39	ag	Xianning, Hubei, CN	Yangtze R.	0.00001	0.00001	0.99998
YW46	bx	Xianning, Hubei, CN	Yangtze R.	0.00001	0.00001	0.99998
YC4	bb	Yichang, Hubei, CN	Yangtze R.	0.00001	0.99998	0.00001
YC5	bc	Yichang, Hubei, CN	Yangtze R.	0.00001	0.99998	0.00001
YC7	bd	Yichang, Hubei, CN	Yangtze R.	0.00001	0.99998	0.00001
YC8	be	Yichang, Hubei, CN	Yangtze R.	0.00001	0.99998	0.00001
YC9	bf	Yichang, Hubei, CN	Yangtze R.	0.00001	0.99998	0.00001
YC10	bg	Yichang, Hubei, CN	Yangtze R.	0.00001	0.99998	0.00001
YC11	bh	Yichang, Hubei, CN	Yangtze R.	0.00001	0.99998	0.00001
YC12	bi	Yichang, Hubei, CN	Yangtze R.	0.00001	0.99998	0.00001
YC15	bj	Yichang, Hubei, CN	Yangtze R.	0.00001	0.99998	0.00001
YC16	bk	Yichang, Hubei, CN	Yangtze R.	0.00001	0.99998	0.00001
YC20	bl	Yichang, Hubei, CN	Yangtze R.	0.00001	0.99998	0.00001
YC24	bm	Yichang, Hubei, CN	Yangtze R.	0.00001	0.99998	0.00001
LZH2	bn	Wuhan, Hubei, CN	Yangtze R.	0.00001	0.00001	0.99998
LZH5	bo	Wuhan, Hubei, CN	Yangtze R.	0.00001	0.00001	0.99998
LZH6	bp	Wuhan, Hubei, CN	Yangtze R.	0.00001	0.00001	0.99998
LZH10	bq	Wuhan, Hubei, CN	Yangtze R.	0.00001	0.00001	0.99998

LZH13	br	Wuhan, Hubei, CN	Yangtze R.	0.00001	0.00001	0.99998
LZH15	bs	Wuhan, Hubei, CN	Yangtze R.	0.00001	0.00001	0.99998
LZH16	bt	Wuhan, Hubei, CN	Yangtze R.	0.00001	0.00001	0.99998
YH1	by	Luoyang, Henan, CN	Yellow R.	0.00001	0.19912	0.80087
YH2	bz	Luoyang, Henan, CN	Yellow R.	0.00001	0.15493	0.84506
YH4	ca	Luoyang, Henan, CN	Yellow R.	0.00001	0.18419	0.81580
YH9	cb	Luoyang, Henan, CN	Yellow R.	0.00001	0.00001	0.99998
YH10	cc	Luoyang, Henan, CN	Yellow R.	0.00001	0.00001	0.99998
YH13	cd	Luoyang, Henan, CN	Yellow R.	0.00001	0.00001	0.99998
YH14	ce	Luoyang, Henan, CN	Yellow R.	0.00001	0.00001	0.99998
YH15	cf	Luoyang, Henan, CN	Yellow R.	0.00001	0.00001	0.99998
YH19	dj	Luoyang, Henan, CN	Yellow R.	0.00001	0.00001	0.99998
YH24	dk	Luoyang, Henan, CN	Yellow R.	0.00001	0.00001	0.99998
YH26	dl	Luoyang, Henan, CN	Yellow R.	0.00001	0.17942	0.82057
YH28	dm	Luoyang, Henan, CN	Yellow R.	0.00092	0.00001	0.99907
WL4	ch	Jiyuan, Henan, CN	Yellow R.	0.00001	0.00001	0.99998
WL5	ci	Jiyuan, Henan, CN	Yellow R.	0.00001	0.00001	0.99998
WL6	cj	Jiyuan, Henan, CN	Yellow R.	0.00001	0.00001	0.99998
WL7	ck	Jiyuan, Henan, CN	Yellow R.	0.00001	0.00001	0.99998
WL18	cl	Jiyuan, Henan, CN	Yellow R.	0.00001	0.00001	0.99998
WL20	cm	Jiyuan, Henan, CN	Yellow R.	0.00001	0.00001	0.99998
WL23	cn	Jiyuan, Henan, CN	Yellow R.	0.00001	0.00001	0.99998
WL24	co	Jiyuan, Henan, CN	Yellow R.	0.00001	0.00001	0.99998
QSH3	cp	Laibing, Guangxi, CN	Pearl R.	0.99998	0.00001	0.00001
QSH4	cq	Laibing, Guangxi, CN	Pearl R.	0.99998	0.00001	0.00001
QSH 8	cr	Laibing, Guangxi, CN	Pearl R.	0.99998	0.00001	0.00001
QSH 9	cs	Laibing, Guangxi, CN	Pearl R.	0.99998	0.00001	0.00001
QSH 10	ct	Laibing, Guangxi, CN	Pearl R.	0.99998	0.00001	0.00001
QSH18	cv	Laibing, Guangxi, CN	Pearl R.	0.99998	0.00001	0.00001
QSH20	cx	Laibing, Guangxi, CN	Pearl R.	0.99998	0.00001	0.00001
LB1	cy	Liuzhou, Guangxi, CN	Pearl R.	0.99998	0.00001	0.00001
LB2	cz	Liuzhou, Guangxi, CN	Pearl R.	0.99998	0.00001	0.00001
LB3	da	Liuzhou, Guangxi, CN	Pearl R.	0.99998	0.00001	0.00001
LB5	db	Liuzhou, Guangxi, CN	Pearl R.	0.99998	0.00001	0.00001
LB6	dc	Liuzhou, Guangxi, CN	Pearl R.	0.99998	0.00001	0.00001
LB7	dd	Liuzhou, Guangxi, CN	Pearl R.	0.99998	0.00001	0.00001
LB8	de	Liuzhou, Guangxi, CN	Pearl R.	0.99998	0.00001	0.00001
LB9	df	Liuzhou, Guangxi, CN	Pearl R.	0.99998	0.00001	0.00001
LB10	dg	Liuzhou, Guangxi, CN	Pearl R.	0.99998	0.00001	0.00001
LB11	dh	Liuzhou, Guangxi, CN	Pearl R.	0.99998	0.00001	0.00001
LB12	di	Liuzhou, Guangxi, CN	Pearl R.	0.99998	0.00001	0.00001
