# Supplementary figures and images for: Population Genomic Evidence for the Diversification of Bellamya aeruginosa in Different River Systems in China
Source: Biology (Basel). 2022 Dec 23;12(1):29. doi: 10.3390/biology12010029 (PMC9855799; doi:10.3390/biology12010029)

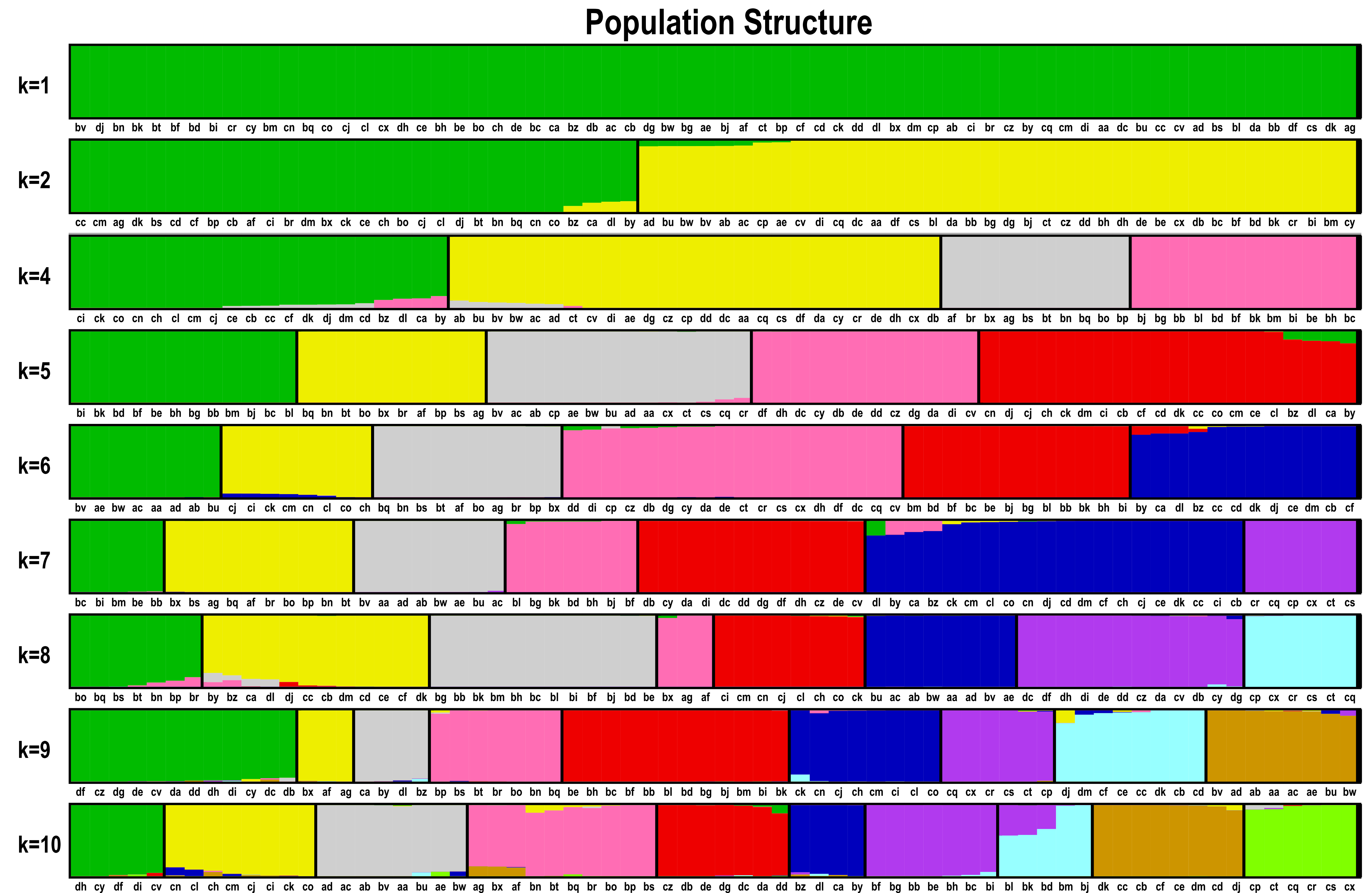

Supplement: Supplementary file 1 [file biology-12-00029-s001.zip › Figure S1.jpg]
